# Supplementary material for: DNA Damage- But Not Enzalutamide-Induced Senescence in Prostate Cancer Promotes Senolytic Bcl-xL Inhibitor Sensitivity
Source: Cells. 2020 Jul 1;9(7):1593. doi: 10.3390/cells9071593 (PMC7408442; doi:10.3390/cells9071593)
Supplement: Supplementary file 1 [file cells-09-01593-s001.zip › 20200618_TIS-Pca_Supp Fig_RevisionFINAL 0709.pdf]

Figure S1

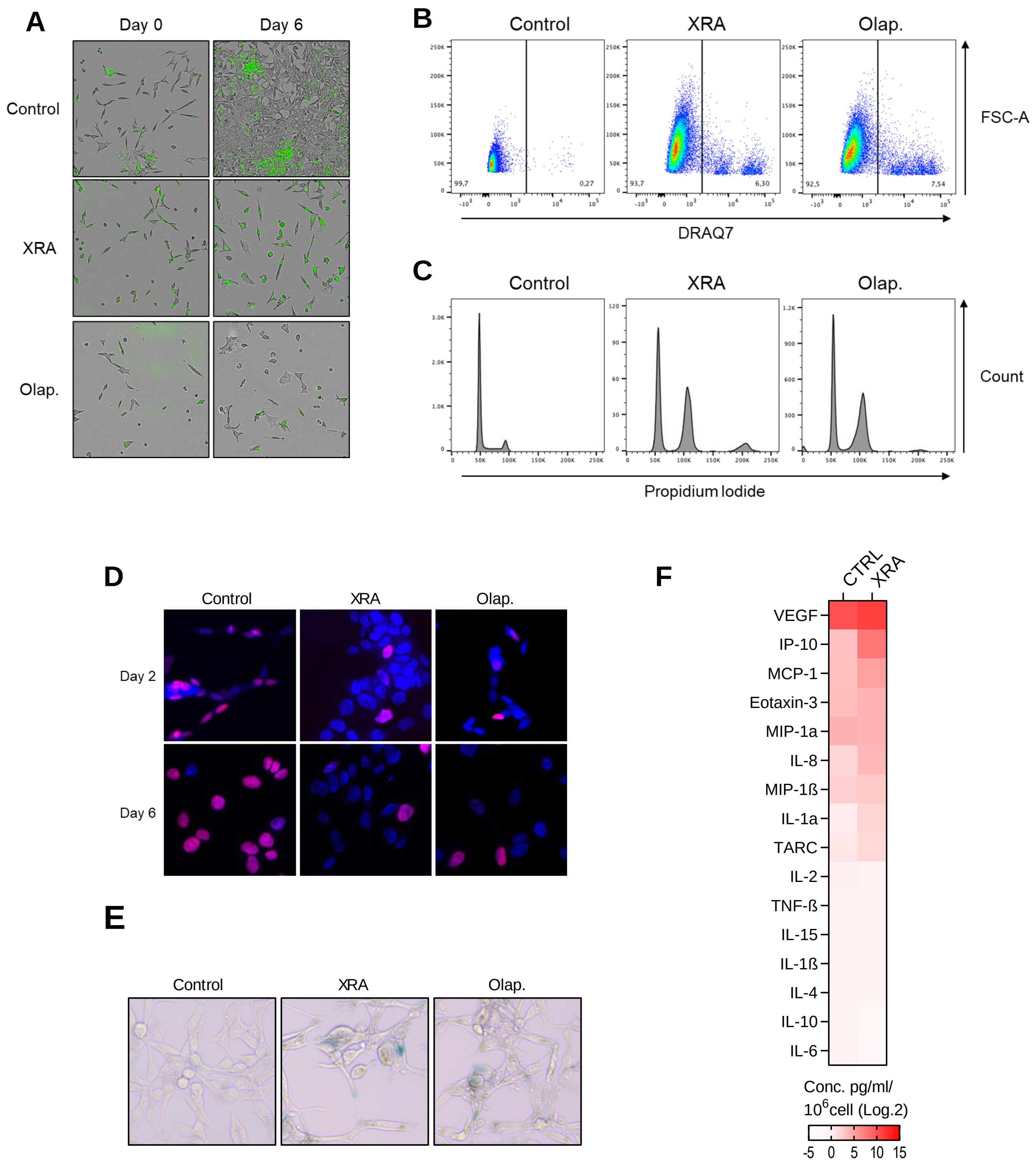

A
Day 0
Day 6
Control
XRA
Olap.
B
Control
XRA
Olap.
99,7
0,27
93,7
6,30
92,5
7,54
FSC-A
DRAQ7
C
Control
XRA
Olap.
Count
Propidium Iodide
D
Control
XRA
Olap.
Day 2
Day 6
E
Control
XRA
Olap.
F
CTRL
XRA
VEGF
IP-10
MCP-1
Eotaxin-3
MIP-1a
IL-8
MIP-1ß
IL-1a
TARC
IL-2
TNF-ß
IL-15
IL-1ß
IL-4
IL-10
IL-6
Conc. pg/ml/
10^6cell (Log.2)
-5 0 5 10 15

Figure S2

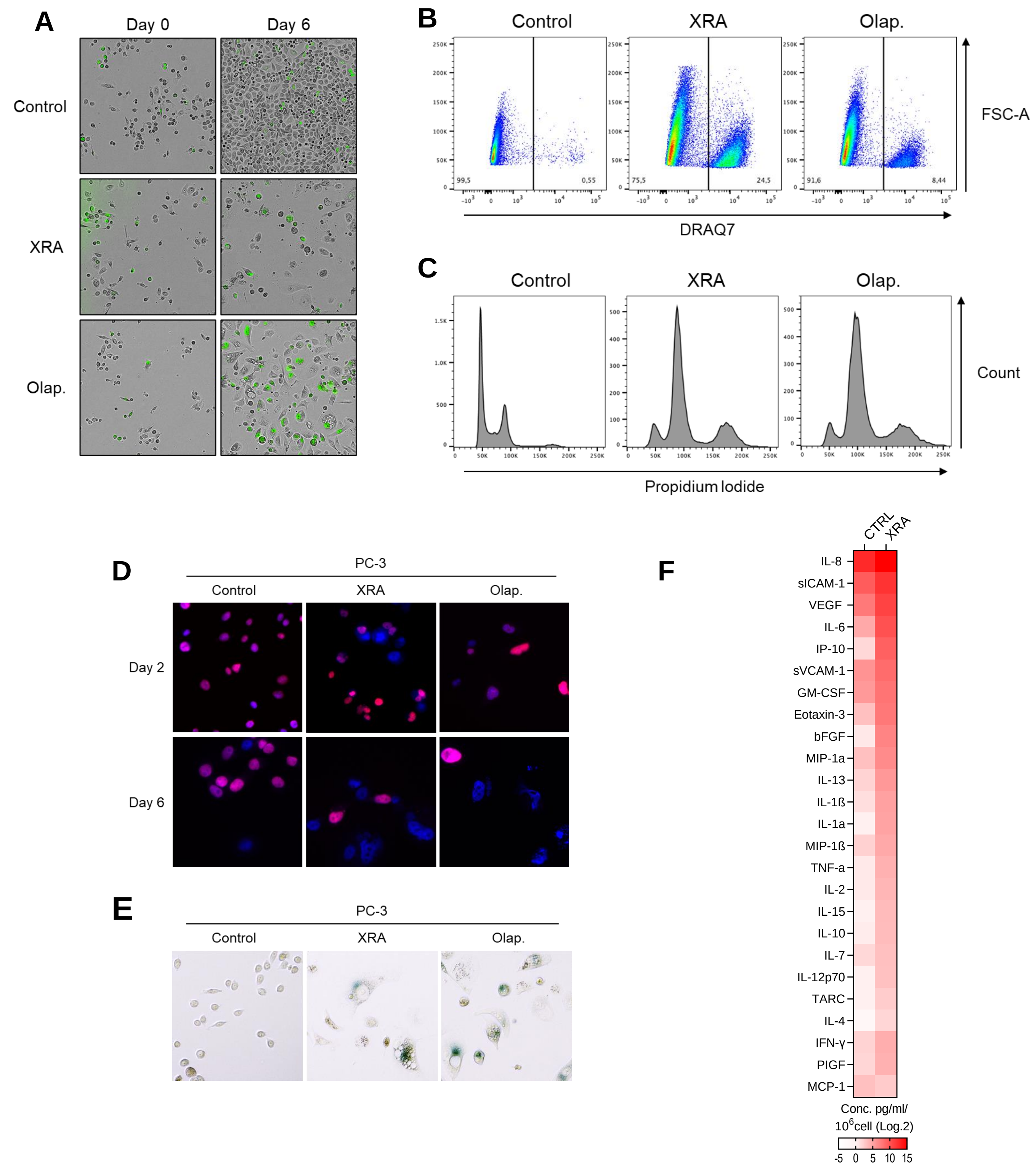
A
Day 0
Day 6
Control
XRA
Olap.
B
Control
XRA
Olap.
99,5
0,55
75,5
24,5
91,6
8,44
FSC-A
DRAQ7
C
Control
XRA
Olap.
Count
Propidium Iodide
D
PC-3
Control
XRA
Olap.
Day 2
Day 6
E
PC-3
Control
XRA
Olap.
F
CTRL
XRA
IL-8
sICAM-1
VEGF
IL-6
IP-10
sVCAM-1
GM-CSF
Eotaxin-3
bFGF
MIP-1a
IL-13
IL-1ß
IL-1a
MIP-1ß
TNF-a
IL-2
IL-15
IL-10
IL-7
IL-12p70
TARC
IL-4
IFN-γ
PIGF
MCP-1
Conc. pg/ml/
10^6cell (Log.2)
-5 0 5 10 15

Figure S3

A

Concentration (µM)

	ABT-263	A115	PPL
S1	0,3125	0,078125	0,15625
S2	0,625	0,15625	0,3125
S3	1,25	0,3125	0,625
S4	2,5	0,625	1,25
S5	5	1,25	2,5

B

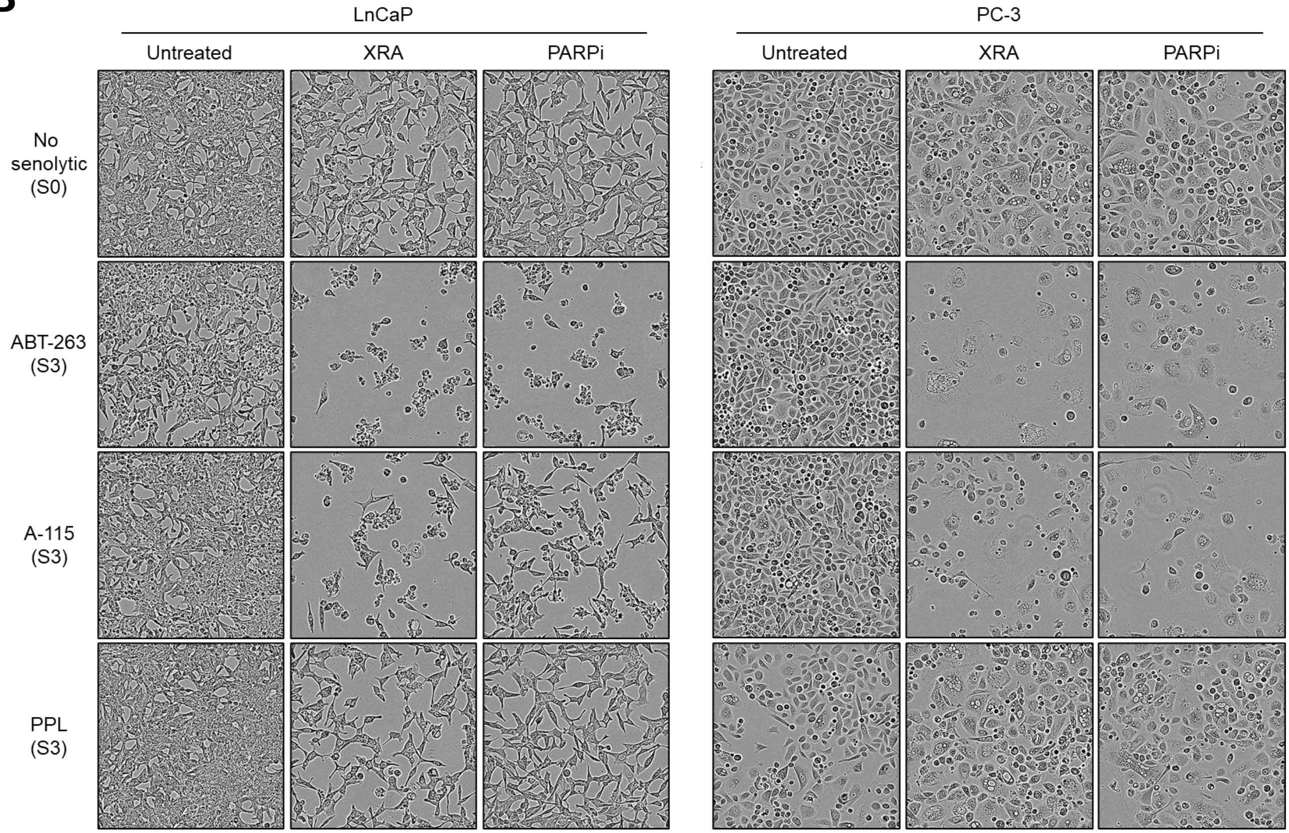

Figure S4

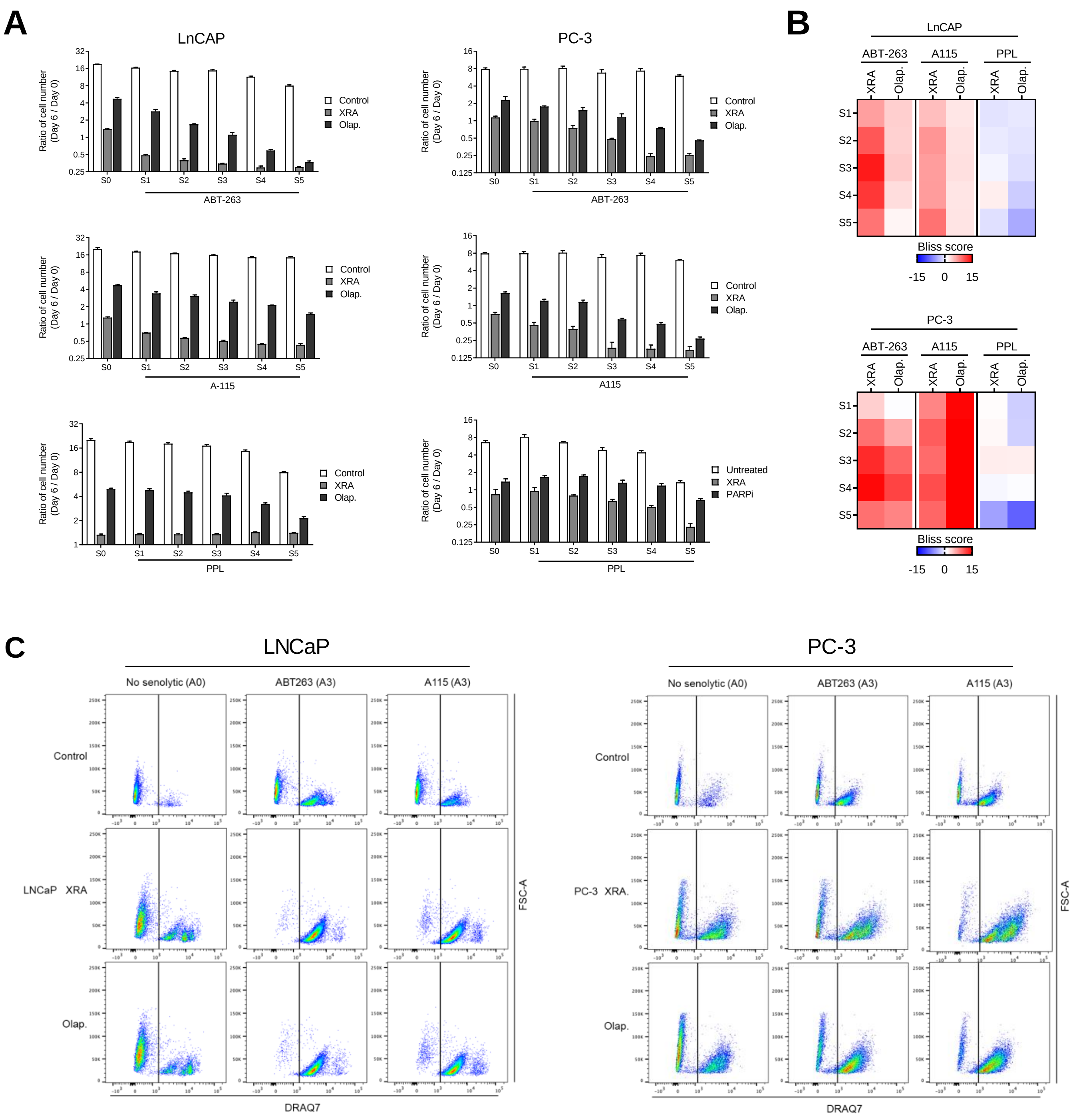
A
LnCAP
PC-3
Ratio of cell number (Day 6 / Day 0)
S0
S1
S2
S3
S4
S5
ABT-263
A-115
A115
PPL
Control
XRA
Olap.
Untreated
PARPi
B
LnCAP
PC-3
ABT-263
A115
PPL
XRA
Olap.
Bliss score
-15
0
15
C
LNCaP
PC-3
No senolytic (A0)
ABT263 (A3)
A115 (A3)
Control
LNCaP XRA
PC-3 XRA.
Olap.
FSC-A
DRAQ7

Figure S5

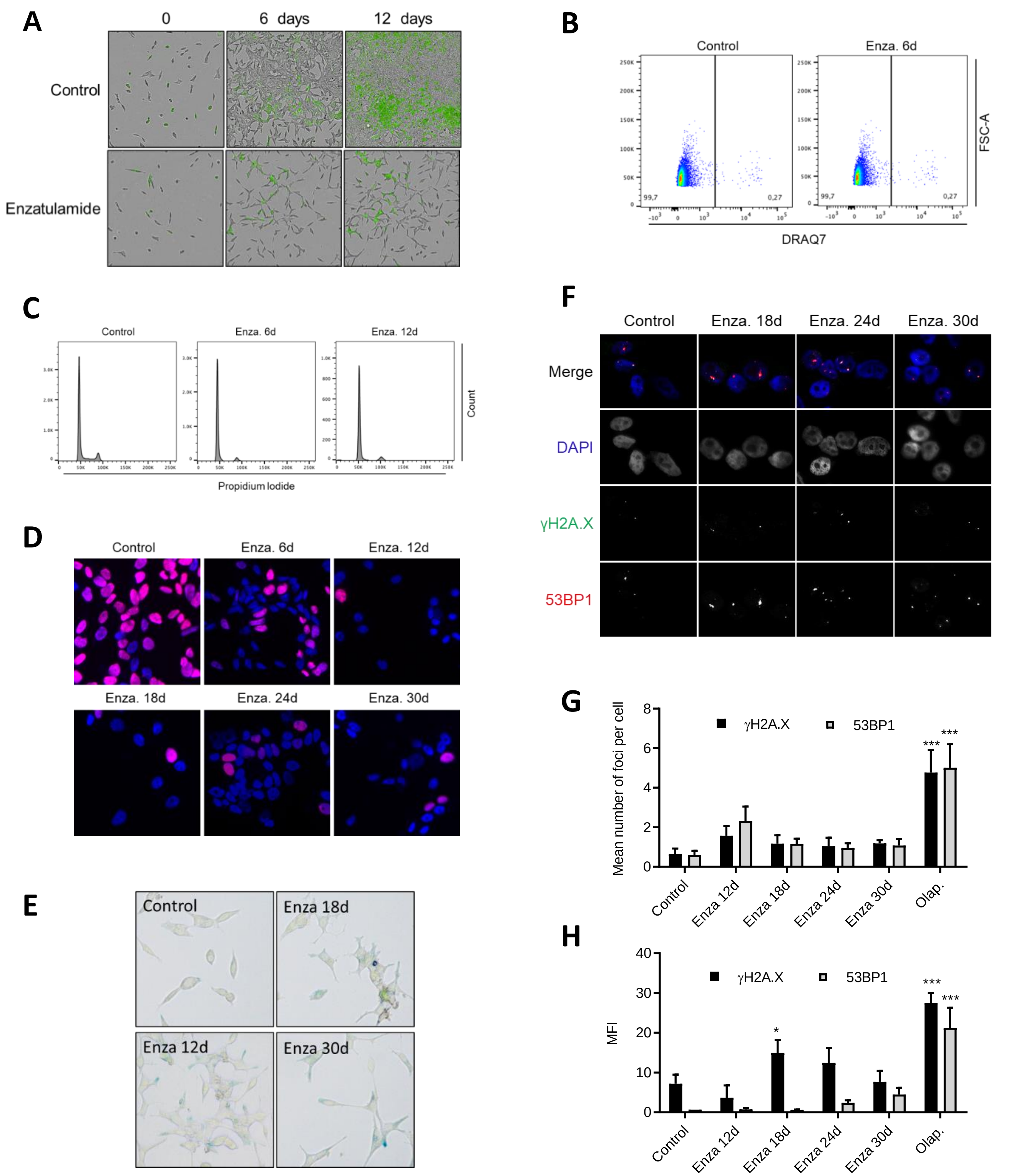
A
0
6 days
12 days
Control
Enzatulamide
B
Control
Enza. 6d
FSC-A
DRAQ7
99,7
0,27
C
Control
Enza. 6d
Enza. 12d
Count
Propidium Iodide
D
Control
Enza. 6d
Enza. 12d
Enza. 18d
Enza. 24d
Enza. 30d
E
Control
Enza 18d
Enza 12d
Enza 30d
F
Control
Enza. 18d
Enza. 24d
Enza. 30d
Merge
DAPI
γH2A.X
53BP1
G
Mean number of foci per cell
γH2A.X
53BP1
Control
Enza 12d
Enza 18d
Enza 24d
Enza 30d
Olap.
H
MFI
γH2A.X
53BP1
Control
Enza 12d
Enza 18d
Enza 24d
Enza 30d
Olap.

Figure S6

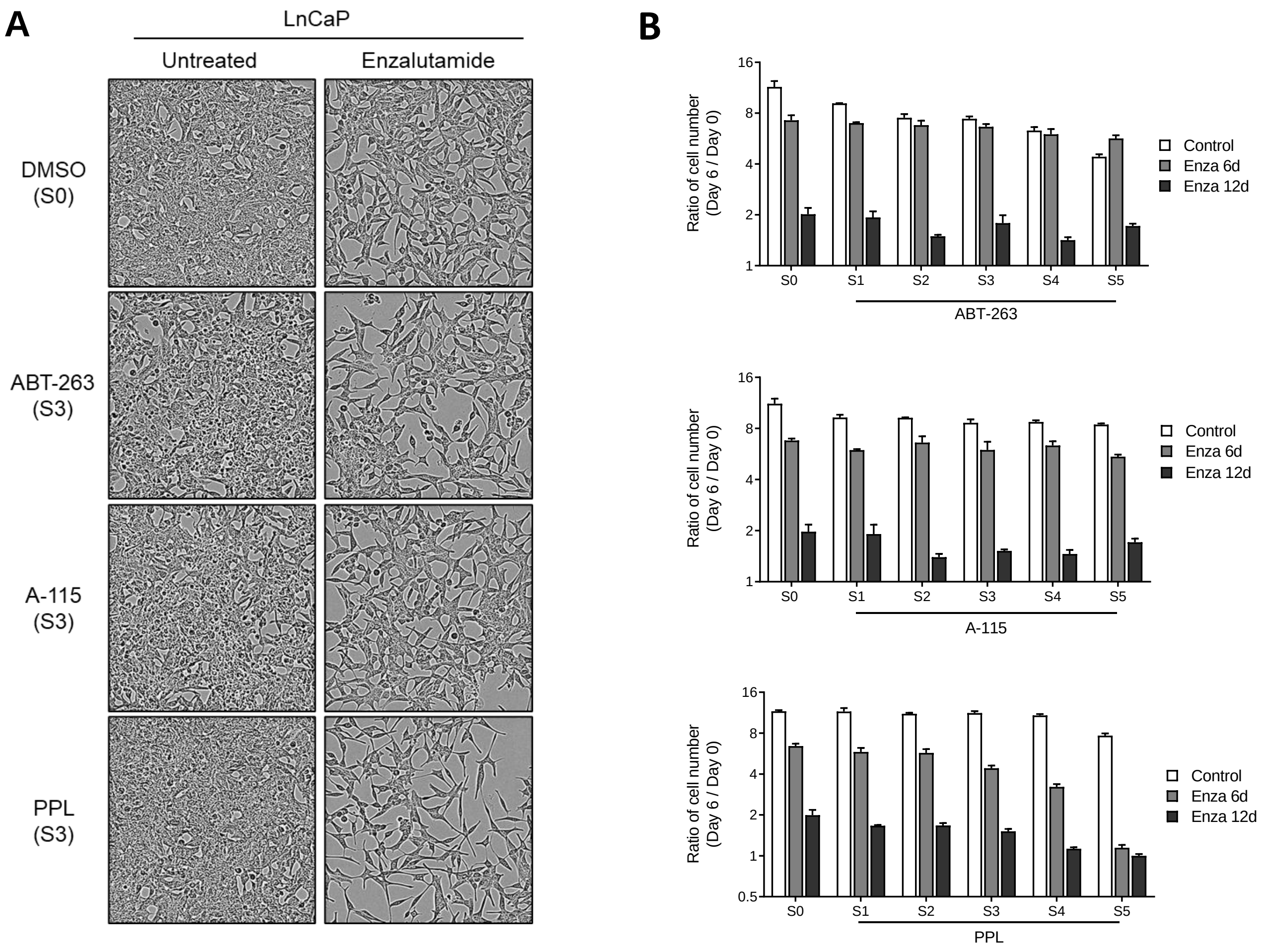
A
LnCaP
Untreated
Enzalutamide
DMSO (S0)
ABT-263 (S3)
A-115 (S3)
PPL (S3)
B
Ratio of cell number (Day 6 / Day 0)
S0
S1
S2
S3
S4
S5
ABT-263
A-115
PPL
Control
Enza 6d
Enza 12d

Table S1

Target	Forward (5'-3')	Reverse (5'-3')
p15	GAATGCGCGAGGAGAACAAG	CATCATCATGACCTGGATCGC
p16	GGGAGCAGCATGGAGCCT	ATGACCTGGATCGGCCTCCGACCGT
p21	GGGACAGCAGAGGAAGAC	TGGAGTGGTAGAAATCTGTCA
p27	TCCGGCTAACTCTGAGGACA	GTAGAAGAATCGTCGGTTGC
p53	GGAAGACTCCAGTGGTAATCTA	TTGGGCAGTGCTCGCTTA
p57	GCGGTGAGCCAATTTAGAGC	CGGTTGCTGCTACATGAACG
IL-8	GCCAACACAGAAATTATTGTAAAG	TTATGAATTCTCAGCCCTCTTC
IL-6	TGTGTGAAAGCAGCAAAGA	GGCAAGTCTCCTCATTGAA
Il-1α	ACCAGTGCTGCTGAAGGAGAT	TCTTAGTGCCGTGAGTTTCCC
IL-1β	AGAAGTACCTGAGCTCGCCA	CTGGAAGGAGCACTTCATCTGT
GM-CSF	CATGTGAATGCCATCCAGGA	CAGGCCCACATTCTCTCACTT
IGFBP-2	GCCCTCTGGAGCACCTCTACT	CATCTTGCACTGTTTGAGGTTGTAC
