# Supplementary material for: DNA Damage- But Not Enzalutamide-Induced Senescence in Prostate Cancer Promotes Senolytic Bcl-xL Inhibitor Sensitivity
Source: Cells. 2020 Jul 1;9(7):1593. doi: 10.3390/cells9071593 (PMC7408442; doi:10.3390/cells9071593)
Supplement: Supplementary file 1 [file cells-09-01593-s001.zip › 20200622_TIS-Pca_Supp FigLegends_final 0709.pdf]

Supplementary figure 1

A) Representative images of LNCaP expressing H2B-GFP (green) at 0 or 6 days after 8 Gy XRA or 5 µM Olap treatment. **B)** The cumulative cell death of LNCaP was analyzed by flow cytometry 6 days after 8 Gy XRA or 5 µM Olap treatment. **C)** Flow cytometry analysis of cell cycle populations after 8 Gy XRA or 5 µM Olap treatment. **D)** Representative images of LNCaP cells that incorporated EdU for 24 hours at 1 or 5 days after 8 Gy XRA or 5 µM Olap treatment. The merged red (EdU) and blue (DAPI) channels show colocalization in purple. **E)** Representative images of SA-ß-gal assay performed on LNCaP cells 6 days after 8 Gy XRA or 5 µM Olap treatment. **F)** Levels of secreted cytokines were measured by MSD serum-based multiplex assay 6 days after 8 Gy XRA.

Supplementary figure 2

A) Representative images of PC-3 expressing H2B-GFP (green) 0 or 6 days after 8 Gy of XRA or 5 µM Olap treatment. **B)** The cumulative cell death of PC-3 was analyzed by flow cytometry 6 days after 8 Gy XRA or 5 µM Olap treatment. **C)** Flow cytometry analysis of cell cycle populations after 8 Gy XRA or 5 µM Olap treatment. **D)** Representative images of PC-3 cells that incorporated EdU for 24 hours at 1 or 5 days after 8 Gy XRA or 5 µM Olap treatment. The merged red (EdU) and blue (DAPI) channels show colocalization in purple. **E)** Representative images of SA-ß-gal assay performed on PC-3 cells 6 days after 8 Gy XRA or 5 µM Olap treatment. **F)** Levels of secreted cytokines were measured by MSD serum-based multiplex assay 6 days after 8 Gy XRA.

Supplementary figure 3

A) Concentrations of ABT-263, A-115 and PPL that were used in senolysis experiments (Figures 3, 5 and 6). **B)** Representative images of LNCaP (left) and PC-3 (right) that were treated for 6 days with 8 Gy of XRA or 5 µM Olap (PARPi) alone or in combination with 0.625 µM ABT-263 (top), 0.3125 µM A-115 (middle) or 0.625 µM PPL (bottom). For all the data, the mean ± SD of three independent experiments is shown. Data were analyzed using the two-tailed Student's t-test.

Supplementary figure 4

(**A-B)** LNCaP or PC-3 expressing H2B-GFP were treated with 8 Gy of XRA or 5 µM Olap for 6 days, alone or in combination with increasing concentrations of ABT-263, A-115 or PPL. **A)** Cell survival histograms and **B)** Bliss scores heat maps for LNCaP and PC-3 that were exposed to the different combination treatments. S0 to S5 correspond to increasing senolytic concentrations (see Figure S3A). (A) Data are the mean ± SEM of triplicate and are representatives of three independent experiments. **C)** Flow cytometry analysis of LNCaP (left) or PC-3 (right) cell death 6 days after 8 Gy XRA or 5 µM Olap treatment alone or in combination with 0.625 µM ABT-263 or 0.3125 µM A-115.

Supplementary figure 5

A) Representative images of LNCaP expressing H2B-GFP (green) at 0, 6 or 12 days following 10 µM Enza treatment. **B)** The cumulative cell death of LNCaP was analyzed by flow cytometry (DRAQ7 staining) 6 days after 10 µM Enza treatment. **C)** Flow cytometry analysis of cell cycle populations following 6 or 12 days 10 µM Enza exposure. **D)** Representative images of LNCaP cells that incorporated EdU for 24 hours, 6, 12, 18, 24 or 30 days following 10 µM Enza exposure. The merged red (EdU) and blue (DAPI) channels show colocalization in purple. **E)**

Representative images of SA-ß-gal assay performed on LNCaP cells following 12, 18 or 30 days of 10 µM Enza exposure. **F)** Representative images of γH2AX (green) and 53BP1 (red) foci per nucleus in LNCaP cells following 6, 12, 18, 24 or 30 days of 10 µM Enza exposure. The merged red and green channels show colocalization in yellow and DAPI is shown in blue. **(G-H)** Quantification of γH2AX and 53BP1 foci G) number or H) mean fluorescence intensity (MFI) per nucleus. Data are the mean ± SEM of triplicate and are representatives of three independent experiments. Two-way ANOVA. * $p < 0.05$, *** $p < 0.001$.

Supplementary figure 6

A) Representative images of LNCaP that were exposed 6 days to 10 µM Enza, alone or in combination with 0.625 µM ABT-263 (top), 0.3125 µM A-115 (middle) or 0.625 µM PPL (bottom). **B)** Cell survival histograms of LNCaP and PC-3 that were treated with 10 µM Enza for 6 or 12 days, alone or in combination with increasing concentrations of ABT-263 (top), A-115 (middle) or PPL (bottom). S0 to S5 correspond to increasing senolytics concentrations (see Figure S3A). Data are the mean ± SD of triplicate and are representatives of three independent experiments.

Supplementary Table 1

Forward and reverse primers that were used to detect CDKi and SASP gene transcripts in Q-PCR experiments.
